# Co-gelation of gluten and gelatin as a novel functional material formation method

**DOI:** 10.1007/s13197-019-04042-8

**Published:** 2019-08-27

**Authors:** Marta Wesołowska-Trojanowska, Marta Tomczyńska-Mleko, Konrad Terpiłowski, Siemowit Muszyński, Katsuyoshi Nishinari, Maciej Nastaj, Stanisław Mleko

**Affiliations:** 1 Department of Biotechnology, Human Nutrition and Food Commodity Science, University of Life Sciences, Skromna 8, 20-704 Lublin, Poland; 2 Institute of Plant Genetics, Breeding and Biotechnology, University of Life Sciences in Lublin, Akademicka Street 15, 20-950 Lublin, Poland; 3 Department of Physical Chemistry-Interfacial Phenomena, Maria Curie Skłodowska University, M. Curie Skłodowska Sq. 3, 20-031 Lublin, Poland; 4 Department of Physics, University of Life Sciences in Lublin, Akademicka Street 13, 20-950 Lublin, Poland; 5 Glyn O. Phillips Hydrocolloids Research Centre, Hubei University of Technology, Wuhan, People’s Republic of China; 6 Department of Milk Technology and Hydrocolloids, University of Life Sciences in Lublin, Skromna 8, 20-704 Lublin, Poland

**Keywords:** Biopolymer, Gluten, Gelatin, Microstructure, Rheology, Surface properties

## Abstract

Gelatin solution was added to the gluten dispersion to obtain 25% protein from gluten and 0, 0.3, 0.6 and 1.0% of gelatin. Heat-induced gels were formed. The gelatin was leached by immersing the gel straps in distilled water at 45 °C for 2 h. Incorporation of gelatin into the gluten gel matrix resulted in its strengthening. Increase in elastic properties with the increasing amount of gelatin was also found for the macerated gels. The tangent delta showed the minimum for the leached gel with the initial concentration of gelatin 0.6%, so probably at this concentration there was some reinforcement of gluten, or the structure of gluten matrix was formed with the best ability to include gelatin inside. FTIR (Fourier transform infrared spectroscopy) results showed, that at the 0.6% gelatin concentration more gelatin was present in the leached samples than in the 1% gelatin added samples. Gelatin gels can act as an active filler reinforcing the gluten microstructure. Leaching of gelatin from the mixed gel matrix resulted in the microstructure with visible phase separation. Generally gelatin addition gave a surface smoothing effect and lower surface roughness of the obtained gels. Pure gluten gels soaking in hot water resulted in the decreased roughness. Possibility of manipulation with gluten gels surface roughness by co-gelling with gelatin can have an influence on the application of such gels as matrices for active ingredients.

## Introduction

Grain storage proteins can be obtained on a large scale and are used for different food applications. The most important are: gluten from wheat and zein from corn. Gluten is a highly elastic mass left when wheat flour is deprived of starch and other polysaccharides. It is composed of ca. 85% protein and 5% lipids, water and residual carbohydrates (Wieser 2007). Gluten enables wheat dough to have unique mechanical properties, such as high elasticity and strain hardening. Hydration and kneading of gluten give high elasticity for a cohesive mass.

Two main fractions of proteins in gluten are: gliadins and glutenins. Glutenins are differentiated into two main fractions: high molecular weight (HMW) (600–800 amino acids) and low molecular weight (LMW) (ca. 300 amino acids). The main crosslinking reaction between the gluten protein chains is thiol–disulfide interchange, as almost all glutenins contain cysteine. Intermolecular or intramolecular bridges are formed depending on the position of this amino acid in the protein chain. For gliadins disulfide bridges are formed mostly inside the protein chain, leading to intramolecular self-crosslinking. Due to this, gliadins increase only viscosity of the gluten without increasing its elasticity (Zielbauer et al. 2017). Elastic properties of gluten are determined by those of glutenins. HMW glutenins have cysteines at the end of molecules which enable formation of intermolecular bridging extending protein chain length forming elastic structures in this way. LMW glutenins having cysteine in different places of protein chain form both inter- and intramolecular bonds. Formation of a three dimensional gluten matrix must be preceded by heating which denatures proteins exposing reactive groups. The sulfhydryl group/disulfide bond interchange is the main reaction responsible for proteins junction. At the kneading of gluten, further interactions take place, mostly hydrophobic and electrostatic ones (Wieser 2007). During heating of wheat gluten dispersions, unfolding of protein chains was observed. Changes in protein structure cause exposition of free sulfhydryl groups and hydrophobic regions. After unfolding, protein chains are aggregated by hydrophobic interactions and disulfide bonds. During the gluten gelation transition towards the β-sheets secondary structures is observed (Wang et al. 2017).

Gelatin originates from the collagen which is present in muscles connective tissues. Collagen is composed of triple helices. It denatures to gelatin which is widely used as a gelling agent. Gelatin gels at the temperatures below 40 °C with partial reversion of random coils form more ordered triple helical structures. The obtained gel matrix is stabilized by the hydrogen and electrostatic bonds with the additional hydrophobic effect (Bello et al. 1962; Tadeo et al. 2009). The gelatin gel is thermoreversible which enables its leaching out from the mixed systems at the temperatures above 40 °C. Shim et al. (2017) obtained a photocrosslinkable hydrogel based on the gelatin/methacrylate. Gelatin playing a role of porogen was leached out of the polymer matrix producing micropores which increased cell viability in the 3D scaffolds. Micropores with a rapid swelling ability enabled an efficient nutrient exchange for bacteria in the 3D scaffold based on cellulose/gelatin (Khan et al. 2016). This kind of scaffold can be used for tissue regeneration. Oechsle et al. (2016) produced collagen films by extrusion with other proteins. They noticed that application of different proteins is a good method for foods textural and sensory properties modification. Mixing several polymers or hydrocolloids allows the development of new materials with desired applications. Interactions between different hydrocolloids can lead to segregation or aggregation depending on their surface charges. According to the Coulomb’s law single-sign charges are repulsive and opposite charges are attractive. Segregation of different hydrocolloids causes phase separation resulting in the decrease in large and small deformation rheological properties (Jara et al. 2010). The gelatin and gluten mixtures were used previously for edible films formation. Fakhouri et al. (2018) obtained films with different of gelatin and gluten concentrations and the addition of different fatty acids.

Recently intensive development on non-food applications of gluten has been observed. Gomez-Martinez et al. (2013) used gluten for obtaining a biodegradable bioplastic capable of active ingredients release. They believe that this kind of material can be very promising for many applications in pharmacy, medicine, packaging and agriculture. Zhang et al. (2010) observed biodegradation of chemically modified natural polymers based on the wheat gluten. The obtained material was completely decomposed within 22 days of storage in soil. Our recent research has been focused on obtaining biodegradable packaging materials based on gluten, globular proteins and aluminosilicates (Wesołowska-Trojanowska et al. 2016, 2017, 2018). It was found that for the mixtures of gluten, whey proteins and aluminosilicates, there is an optimal concentration of gluten for obtaining a good material for containers shaping. Moreover, gluten is the main material responsible for their elastic properties, although gluten itself without any additives is not a good material for shaping. It is too coherent and elastic (Wesołowska-Trojanowska et al. 2016). These properties depend on very long gluten polymer chains (Wieser 2007).

There are no papers on the mixed gluten/gelatin gels in the scientific literature. Co-gelation of gluten and gelatin with subsequent leaching of gelatin could lead to formation of a tailored gluten matrix with changed viscoelastic, textural and surface properties.

## Materials and methods

Wheat gluten (WG) powder was supplied by Massive (Czechowice-Dziedzice, Poland). According to the supplier, the powder contained 84.1% protein with 7.8% starch, 1.51% fat and 0.69% ash. Gelatin from the bovine bone (lot STH0064) was purchased from Wako Pure Chemical Industries (Osaka, Japan).

### Preparation of heat-set gluten/gelatin gels

The dispersion of gluten (25% in the final mixture) was prepared in distilled water by stirring for 10 min with the magnetic stirrer. The 5% gelatin solution in the distilled water was prepared by heating for 15 min at 80 °C using an electric heater with magnetic stirring. The gelatin solution was added to the gluten dispersion to obtain 25% of gluten and 0, 0.3, 0.6 and 1.0% of gelatin. The mixture was stirred for 10 min using a glass baguette and heated for 30 min at 80 °C. Immediately after heating the samples were cooled with cold tap water to 20 °C to form a gelatin gel. The samples were cut into 5 mm thick straps using a wire cutter. The gelatin was leached from some samples by immersing the straps in distilled water at 45 °C for 2 h. The samples were stored at 6 °C for 18 h before the analysis.

### Small strain rheology

The dynamic oscillatory measurements were conducted using the RS300 (ThermoHaake, Karlsruhe, Germany) rheometer equipped with a serrated parallel steel plate geometry (35 mm diameter, 2 mm gap size) to eliminate potential sliding effects. For the rheological analysis discs with a 15 mm diameter and 5 mm thickness were cut using a cork borer. The samples were analyzed using the frequency sweeps in the range 0.1–10 Hz. All measurements were performed in the linear viscoelastic region (at 1% strain) which was evaluated by strain sweeps. The temperature of all measurements was 21 °C.

### Large strain rheology

Large strain compression tests were carried out using an XT.T2 Texture Analyzer (Stable Micro Systems, Surrey, U.K.). For the uniaxial compression the samples of a 15 mm diameter and 5 mm thickness were compressed to 50% strain at the 0.5 mm/s plunger rate. The samples were compressed using a compression platen P/100. Force and distance to break were determined and were treated as hardness and elasticity (Bourne 2002).

### Ultrasound viscosity measurements

The probe was immersed into the gel and the values of viscosity × density (mPas × g/cm^3^) were measured. All the measurements were made using an ultrasound viscometer Unipan type 505 (UNIPAN, Warsaw, Poland) equipped with a magnetostrictive probe (47 mm long). Six measurements were performed to obtain a single average result.

### Scanning electron microscopy (SEM)

The biopolymers were dried at 45 °C in a laboratory drier for 24 h. The samples were fixed in the 2.5% solution of glutaraldehyde in the 0.1 M sodium cacodylate buffer. The biopolymers were dehydrated in the serial dilutions of ethanol and acetone. Then they were dried at the critical point in liquid carbon dioxide. The samples were coated with pure gold in a vacuum evaporator EMITECH K550x (Emitech, Ashford, United Kingdom). The biopolymers were observed in a scanning electron microscope JEOL JXA-8230 (Tokyo, Japan).

### Surface roughness

The surface roughness of the dried biopolymer was measured using an optical profilometer—GT Contour Surface Metrology (Veeco, Tucson, USA). The roughness coefficients were determined using the Vision64 computer program (Veeco, Tucson, USA).

### Fourier transform infrared spectroscopy (FTIR)

The FTIR spectra of the samples were recorded on the Nicolet iS10 research-grade FTIR spectrometer (Thermo Fisher Scientific, Waltham, U.S.A.) using the Attenuated Total Reflectance (ATR) mode. The spectral range was 4000–400 cm^−1^, the resolution was set to 4 cm^−1^.

### Statistical analysis

The statistical analysis was performed using the statistical program STATISTICA 5.0 PL (StatSoft Polska, Warsaw, Poland). The analysis of variance was made and the standard deviation was calculated. The significance of differences between the mean values was determined using the Tukey’s test at the confidence level of *P* ≤ 0.05.

## Results and discussion

### Rheological properties

**Fig. 1:**
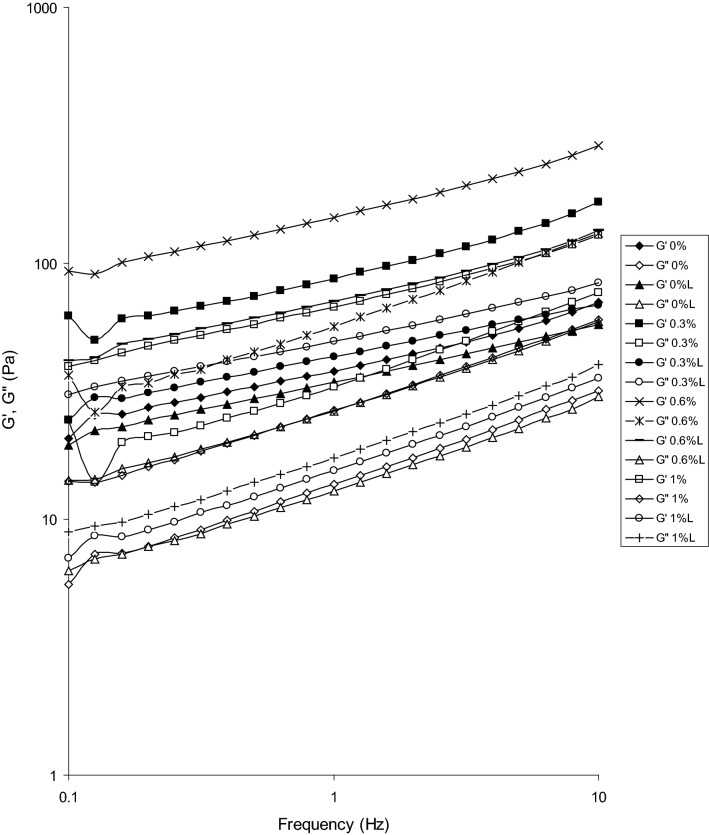
Influence of frequency on the storage and loss moduli for the mixed gluten/gelatin (%) unleached and leached (L) gels

Figure 1 shows the frequency sweeps of the viscoelastic properties of the obtained biopolymers. The increased frequency resulted in the increasing values of storage and loss moduli. At higher frequency the material had less time for relaxation and according to the Debora law it behaved as more rigid, elastic. The increased gelatin concentration up to 0.6% resulted in the increased moduli values. A further increase of gelatin concentration (1.0%) did not cause a further increase in the storage and loss moduli. The leaching process led to formation of a less rigid matrix with smaller values of the moduli. Incorporation of gelatin into the gluten gel matrix resulted in its strengthening. More elastic structure was formed which is justified as gelatin forms very elastic gels (Tomczynska-Mleko et al. 2014a, b). Table 1 shows the values of storage modulus and loss tangent at the highest frequency value: 10 Hz. The best elastic properties of the obtained gels with the highest storage modulus and the lowest value of loss tangent at 10 Hz were observed for the samples with 0.6% gelatin added. It is interesting to see that there was an increase in elastic properties of the macerated gels, also up to 0.6% gelatin added. A small addition of gelatin (0.3%) caused an increase in the storage modulus and a decrease in the loss tangent in comparison to the gluten gels. Maceration of the gel with 0.3% gelation resulted in a higher storage modulus but a very similar loss tangent in comparison to the gluten gels. The macerated samples with 0.3% gelatin were more rigid as they had higher storage and loss moduli (Fig. 1), but their relative elasticity was similar (i.e. tangent δ) (Table 1). The increase in the elastic properties with the increasing gelatin for the samples macerated in water can be explained by the fact that leaching does not remove the whole gelatin content. The tangent delta shows the minimum for the leached gel with an initial concentration of gelatin 0.6%, so probably at this concentration there was some reinforcement of the gluten protein matrix, or the structure of gluten matrix was formed with the best ability to include gelatin (Table 1). Both proteins contain cysteine and can form inter- and intramolecular disulfide bonds (Moll and Wagner 1989; Zielbauer et al. 2017). Their formation creates two competing effects: the first one gives longer chains and the second one increases the number and/or molecular weight of filler particles. Both effects will change viscoelastic properties of the gluten/gelatin mixed gels. The gluten powder contained 7.8% of starch, and possible interactions between the gluten, starch and gelatin should be taken also into account. Particularly, it is interesting that maceration of the gluten gel without the added gelatin resulted in the decreased storage modulus and the increased loss tangent (Table 1). This could be explained by washing starch out from the gluten gel matrix.

**Table 1:** Rheological properties of the obtained gels

Sample	G′ at 10 Hz	tan δ at 10 Hz	Max. compr. force (N)	Distance at break (mm)	Ultrasound viscosity (mPas g cm^−3^)
0%	70.43	0.452	0.54 ± 0.03^c^*	1.29 ± 0.03^b^	130 ± 11^b^
0%L	57.73	0.520	0.42 ± 0.02^a^	1.21 ± 0.04^a^	95 ± 14^a^
0.3%	173.5	0.443	0.64 ± 0.02^d^	1.48 ± 0.05^c^	228 ± 18^d^
0.3%L	68.5	0.521	0.48 ± 0.01^b^	1.46 ± 0.05^c^	140 ± 10^b^
0.6%	287.4	0.455	0.72 ± 0.01^e^	1.73 ± 0.04^d^	286 ± 20^e^
0.6%L	134.6	0.438	0.58 ± 0.02^c^	1.44 ± 0.08^c^	178 ± 11^c^
1.0%	129.1	0.467	0.70 ± 0.03^e^	1.14 ± 0.07^a^	162 ± 18^bc^
1.0%L	83.84	0.478	0.58 ± 0.03^c^	1.18 ± 0.05^a^	158 ± 13^bc^

*The means within a column followed by various letters (a–e) are significantly different (*P* ≤ 0.05)

The maximum force and deformation at the fracture of the gels point out to hardness and elasticity, respectively (Bourne 2002). The obtained gels were harder and more elastic with the increasing gelatin content as the maximum force and distance at break in the compression test increased (Table 1). Similarly to the small strain rheology data, leaching caused a decrease in hardness and elasticity but still there was an increase in these properties with the increasing gelatin content. Also maximalization of these properties was observed for the samples with 0.6% gelatin concentration. The samples with 0.3% gelatin had the properties between 0% and 0.6% gelatin concentration samples. This finding is supported by another method of analysis—the ultrasound viscosity measurement (Table 1) which is the other small strain rheological method. It is based on damping of ultrasound vibrations generated in a metal probe. The maximum values of viscosity were noted for the samples with the 0.6% gelatin addition. The increased gelatin content in the composite films with the soy protein isolate caused an increase in tensile strength, elongation to break and elastic modulus (Cao et al. 2007). Investigating the gelatin/gluten films Fakhouri et al. (2018) observed a decrease in elongation at break and an increase in tensile strength with the increasing gelatin content. A higher proportion of gelatin in the films produced less elastic, but more rigid material. In our research the addition of small quantities of gelatin to the gluten matrix caused that the obtained mixed system was harder and more elastic. This contradiction to the findings by Fakhouri et al. (2018) is easy to explain as their samples were composed of very different proportions of gluten and gelatin (from 4:1 to 1:4). Our preliminary attempts showed that this kind of material is characterized by very good shaping properties and can be used for numerous applications as new packaging material or a medical scaffold.

In our research gelatin gel can act as an active filler reinforcing the gluten microstructure. Theoretically leaching should lead to removal of gelatin from the gluten microstructure but very strong insoluble gluten could include gelatin micro gels inside its matrix. The glutamic acid content in gluten is about 40% of the total protein and that of basic amino acids is very low (Tujioka et al. 2011). This results in low total positive charges on the gluten surface. Weak repulsion forces in gluten help dough formation and small positive charges enable interactions with other hydrocolloids with the net negative charges. The surface isoelectric point of gluten is close to pH 7.5 and for gelatin it is 4.7 (Tschoegl and Alexander 1960; Hitchcock 1924). This indicates that the interactions between gluten and gelatin are possible and can lead to the aggregation process. This is the confirmation of increased hardness and storage modulus of the mixed gluten/gelatine gels after leaching out the gelatin. The obtained complexes of these two components are probably less susceptible to leaching than pure gelatin.

### Scanning electron microscopy

**Fig. 2:**
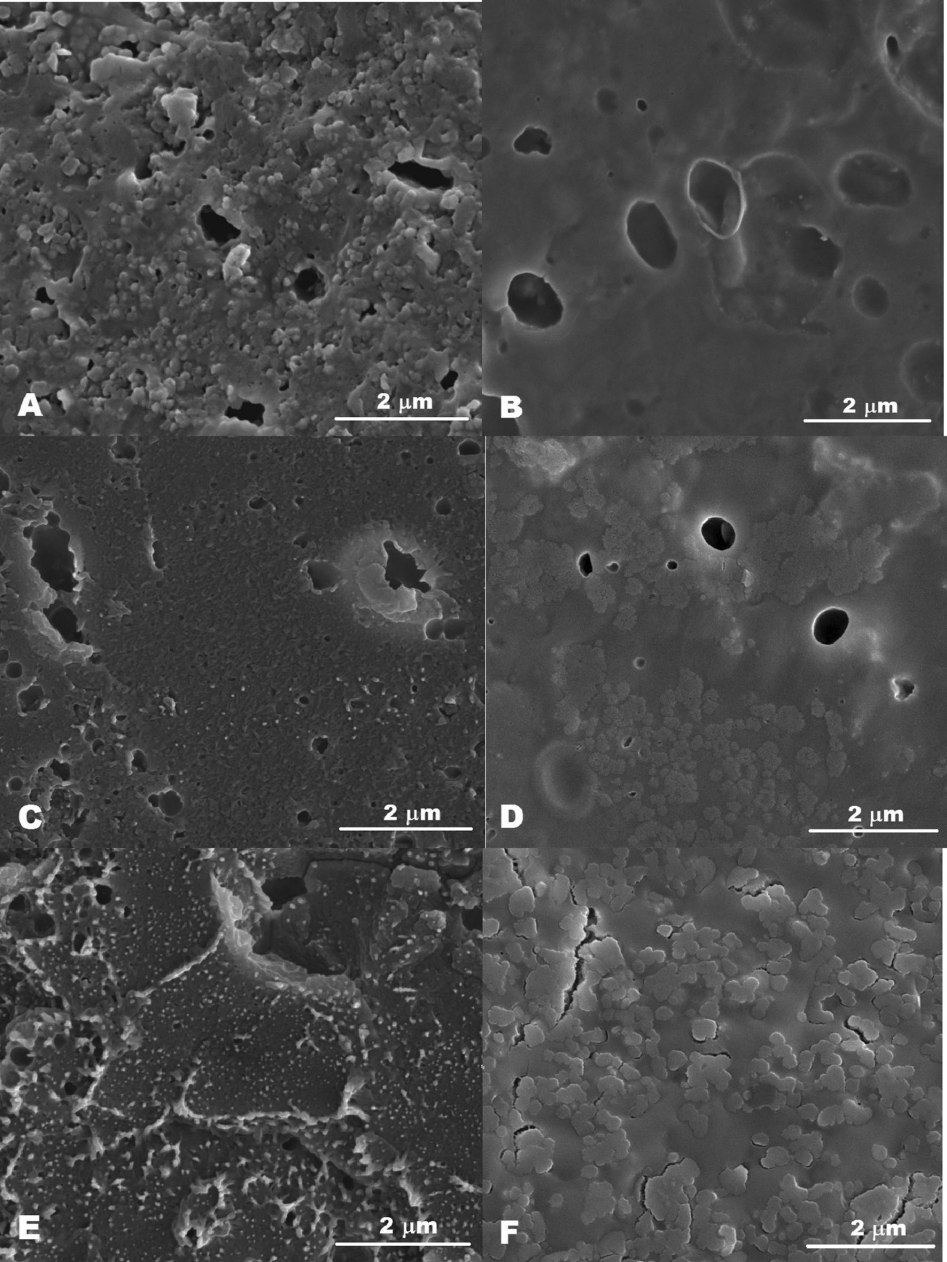
SEM images of gels with 25% gluten and: **a** without gelatin; **b** without gelatin, leached; **c** with 0.6% gelatin; **d** with 0.6% gelatin, leached; **e** with 1.0% gelatin; **f** with 1.0% gelatin, leached

Figure 2 shows the SEM images of gluten with gelatin for the leached and unleached samples. The microstructure of unleached 25% gluten gel was the most homogeneous and the particles of gluten aggregates could be observed. Bonet et al. (2006) noticed a discontinuous and heterogeneous matrix composed of wheat flour and gelatin. The gelatin content was 20% of gluten. In our research the microstructure presented in Fig. 2 contained 2.4 or 4.0% of gelatin compared to gluten. A more heterogeneous microstructure observed by Bonet et al. (2006) was due to other flour ingredients than gluten (e.g. starch). Leaching of gluten gel caused smoothening of the microstructure (Fig. 2b) which probably resulted in a weakened texture with lower hardness, viscosity, rheological moduli, and higher tan delta (Table 1, Fig. 1). The addition of gelatin into the gluten matrix resulted in microstructure changes. A continuous microstructure of unleached structure was observed for both 0.6% and 1.0% gelatin (Fig. 2c, e). Leaching of gelatin from the mixed gel matrix gave a microstructure with visible phase separation. This phenomenon is more visible for the 1% gelatin addition (Fig. 2d, f). The sample with 0.6% of gelatin had a higher value of storage modulus and lower loss tangent which was probably due to a more concise microstructure (Figs. 1, 2d). Although phase separation is observed for the leached samples with gelatin, the structure is harder and more elastic than that of gluten without gelatin. This is probably due to the interactions between gluten and gelatin and the fact that gelatin was not removed entirely from the gluten gel matrix in the leaching process.

### Surface roughness

**Fig. 3:**
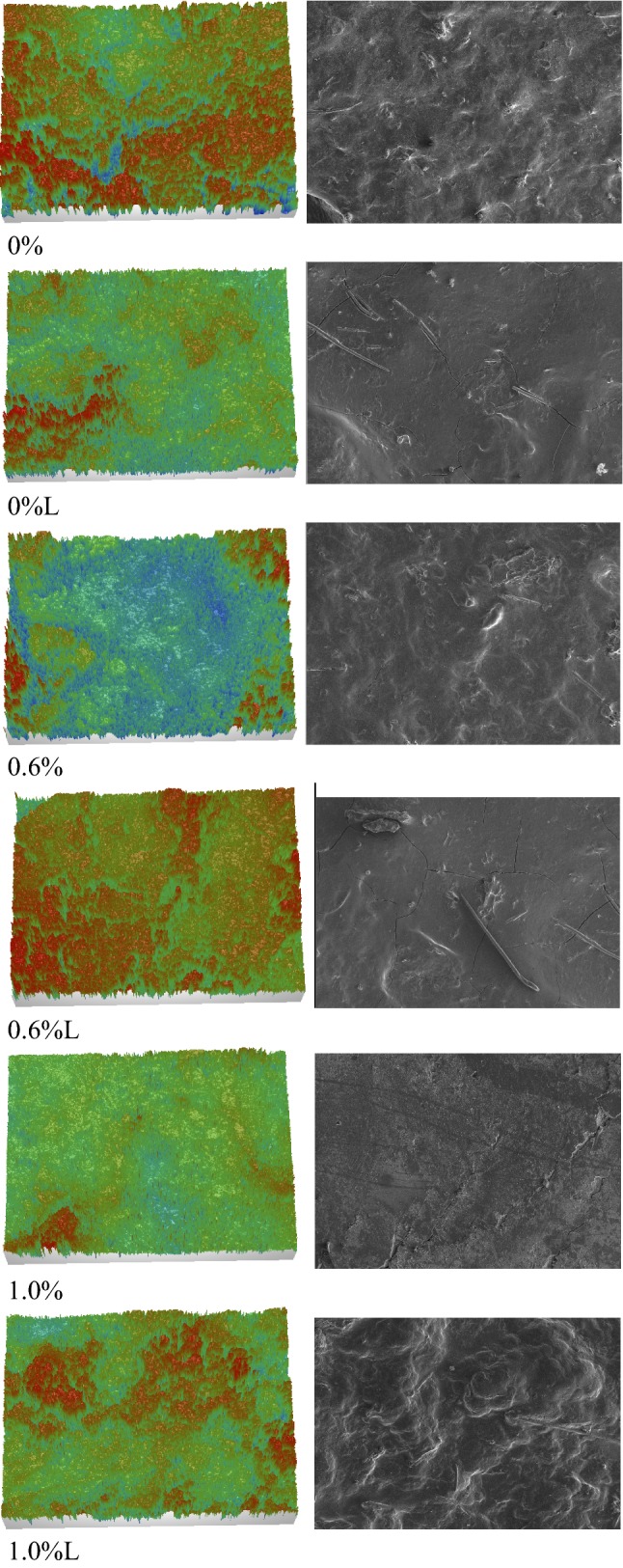
3D profilometer images and SEM pictures of the surface of mixed gluten/gelatin (%) unleached and leached (L) gels. The size of both images is the same: 1300 × 900 μm which is the size for the surface roughness calculation

Using an optical profilometer it is possible to measure roughness of a surface with a very high accuracy. This is a non-contact technique which uses a light source to measure unevenness on the surface in the 3D mode. The method is very useful for soft surfaces like gels as the surface is not destroyed in the measurement process. The obtained images are analyzed by a computer program and different roughness parameters can be calculated: Ra—the average roughness, which is an arithmetic mean of the heights calculated over the whole measured area, Rq—the square root of the heights mean square, Rt—the distance between the highest peak and the lowest valley on the measured surface. Rq parameter is the most representative, as it is independent of local surface heterogeneity. Figure 3 presents the 3D images obtained from the optical profilometer and the SEM surfaces of the same size as scanned by the profilometer (1300 μm × 900 μm). The highest and similar roughness was noted for the gels obtained from gluten with 1.0% of gelatin after its leaching (Table 2). The lowest roughness was observed for the unleached gluten gels with the highest, 1.0% gelatin addition. The smooth gelatin gel acted as the filler of gluten rough gels. The aggregated microstructure of gluten resulted in higher roughness of the surface (Figs. 2a, 3a). The gelatin addition generally resulted in the surface smoothing effect. Similar smoothing of the surface was observed by Nayebzadeh et al. (2006) for polysaccharides co-gelled with the rough heat-induced whey protein gel. Soaking of pure gluten gels in hot water resulted in decreased roughness as the surface was softened and after drying it formed a smoother surface. It seems that this effect was contrary to the process of forming pores by leaching gelatin from the gluten gel and these two effects balanced each other to result in unchanged roughness after leaching. Only the highest concentration of gelatin (1.0%) caused an increase in surface roughness after leaching (Table 2). Possibility to manipulate with gluten gels surface roughness by co-gelling with gelatin can have an influence on the application of such gels as matrices for active ingredients (Mleko et al. 2010; Tomczynska-Mleko et al. 2014a, b; Tomczynska-Mleko 2015). Tomczyńska-Mleko and Mleko (2014) found that the gels microstructure with different surface roughness influenced their contact area with pepsin in an artificial stomach. It caused different release time of active ingredients from the gel matrix. Lately our research group have found that activation of glass support can influence surface properties and also roughness of ion-inducted whey protein gels (Terpiłowski et al. 2017).

**Table 2:** Surface roughness parameters of the obtained gels

Sample	R_a_ (µm)	R_q_ (µm)	R_t_ (µm)
0%	37.9 ± 2.4^a^*	46.3 ± 2.9^a^	352.3 ± 15.1^a^
0%L	27.5 ± 1.7^b^	34.9 ± 2.3^b^	279.7 ± 3.6^c^
0.3%	26.9 ± 1.9^b^	33.4 ± 2.7^b^	271.0 ± 3.7^c^
0.3%L	28.2 ± 2.0^b^	35.6 ± 3.0^b^	237.6 ± 6.6^d^
0.6%	26.7 ± 2.1^b^	34.4 ± 1.3^b^	295.8 ± 10.4^b^
0.6%L	29.9 ± 1.9^b^	36.9 ± 2.3^ab^	313.4 ± 8.9^b^
1.0%	20.0 ± 2.8^c^	25.9 ± 2.1^c^	237.7 ± 4.4^d^
1.0%L	36.0 ± 3.5^a^	43.7 ± 4.7^a^	306.7 ± 5.0^b^

*Ra* the average roughness (an arithmetic mean of the heights), *Rq* the square root of the heights mean square, *Rt* the distance between the highest peak and the lowest valley on the measured surface

*The means within a column followed by various letters (a–d) are significantly different (*P* ≤ 0.05)

### Fourier transform infrared spectroscopy (FTIR)

**Fig. 4:**
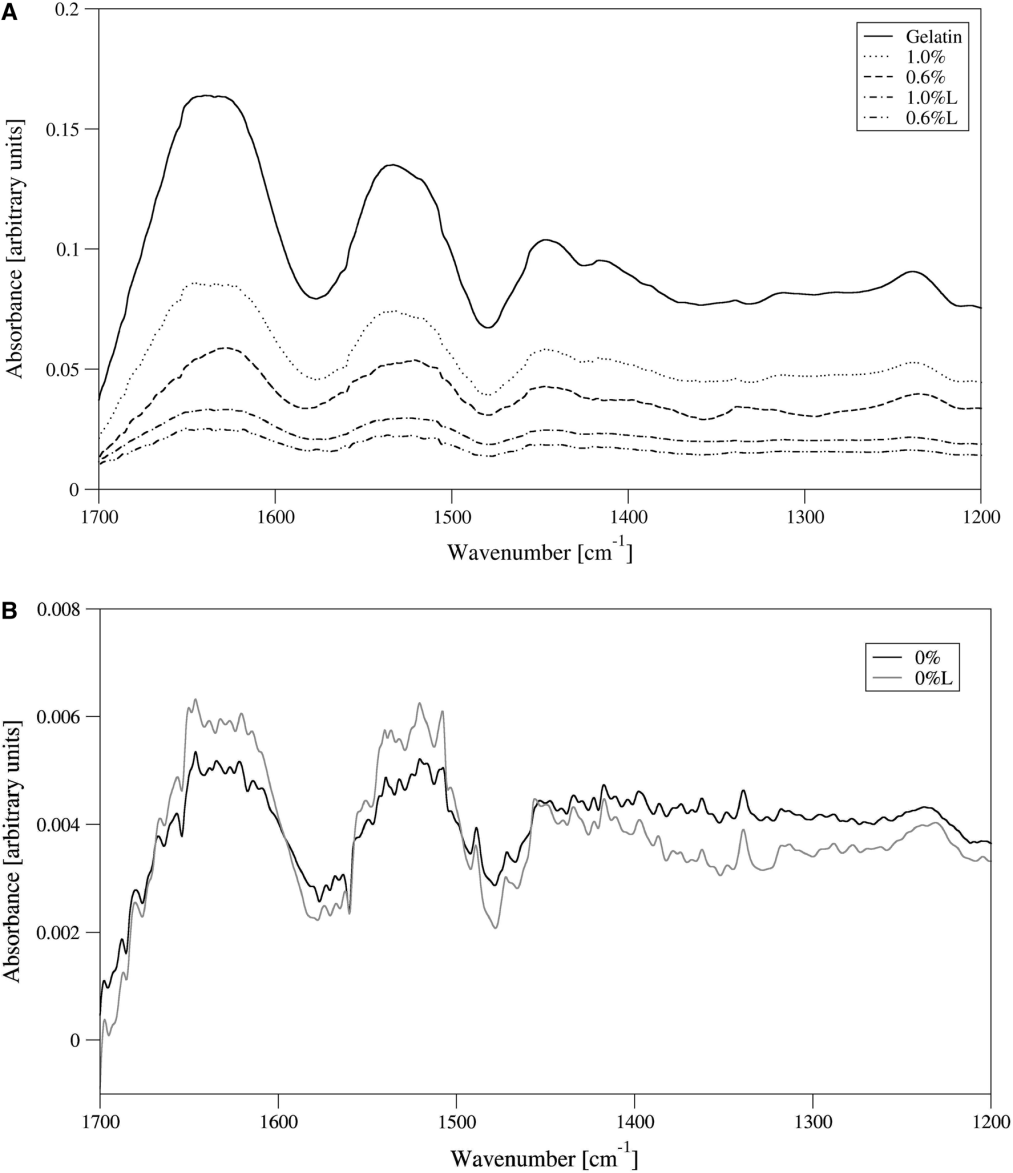
FTIR spectra for **a** the pure gelatin and gluten/gelatin (%) unleached and leached (L) gels; **b** the unleached and leached (L) 25% protein gluten gels

In the pure gelatin FTIR spectrum there is a very weak peak at 1743 cm^−1^ band, and very strong and smooth peaks related to C=O stretching at 1630 cm^−1^ (amide I), N–H bending at 1530 cm^−1^ (amide II) and C–N stretching at 1230 cm^−1^ (amide III) (Fig. 4a) (Etxabide et al. 2015). In the pure gluten FTIR spectrum there is a peak at 1743 cm^−1^ band due to carbonyl containing species in gluten as well as those related to C=O stretching at 1630 cm^−1^ (amide I), N–H bending at 1530 cm^−1^ (amide II) are smaller and not smooth (Fig. 4b) (Chen et al. 2015). The peak responsible for C–N stretching at 1230 cm^−1^ (amide III) is much smaller than in pure gelatin. Soaking of pure gluten gels in warm water does not change the shape of the bands in the FTIR spectra (Fig. 4b). For the unleached samples with gelatin added to gluten, the spectra are similar to the gelatin one, but the absorbance intensity is smaller (Fig. 4a). Leaching decreases the absorbance intensity for both 0.6% and 1.0% added gelatin samples but the shapes of amide I and amide II bands are different. For 0.6% added gelatin the shape is smoother and similar to the pure gelatin sample and for the added 1.0% gelatin, the band contains many small peaks which are similar to the pure gluten sample spectra (Fig. 4b). This proves that at the 0.6% gelatin concentration more gelatin is present in the leached samples than in the 1% gelatin samples. This is probably due to a more concise microstructure of the gel with the 0.6% gelatin concentration in comparison to 1% gelatin gels (Fig. 2c, e). It is more difficult to remove gelatin from more concise microstructure. This confirms our findings that at the 0.6% gelatin concentration the obtained gels are more elastic, harder and have higher viscosity measured by an ultrasound method than in the case of 1.0% gelatin content (Table 1).

## Conclusion

Gelatin gels can act as an active filler reinforcing the gluten microstructure. Leaching should lead to removal of gelatin from the gluten microstructure but very strong insoluble gluten could include gelatin micro gels inside its matrix. The gelatin addition generally resulted in a surface smoothing effect and the obtained gels had lower surface roughness. Soaking of pure gluten gels in hot water resulted in decreased roughness. Possibility to manipulate with gluten gels surface roughness by co-gelling with gelatin can have an influence on many applications of such gels. New functional materials could be created with the applications as scaffolds and drug delivery systems in medicine. The increased roughness enhances the ability of the gastrointestinal enzymes to decompose the gels. Active ingredients can be better adsorbed on the active packaging system surface.
